# The Cerebellum Is Related to Cognitive Dysfunction in White Matter Hyperintensities

**DOI:** 10.3389/fnagi.2021.670463

**Published:** 2021-06-23

**Authors:** Shanshan Cao, Jiajia Nie, Jun Zhang, Chen Chen, Xiaojing Wang, Yuanyuan Liu, Yuting Mo, Baogen Du, Yajuan Hu, Yanghua Tian, Qiang Wei, Kai Wang

**Affiliations:** ^1^The School of Mental Health and Psychological Sciences, Department of Neurology, The First Affiliated Hospital of Anhui Medical University, Anhui Medical University, Hefei, China; ^2^Collaborative Innovation Center of Neuropsychiatric Disorders and Mental Health, Hefei, China; ^3^Anhui Province Key Laboratory of Cognition and Neuropsychiatric Disorders, Hefei, China; ^4^Department of Neurology, The Second Affiliated Hospital of Anhui Medical University, Hefei, China; ^5^Institute of Artificial Intelligence, Hefei Comprehensive National Science Center, Hefei, China

**Keywords:** cerebellum, white matter hyperintensities, resting-state functional connectivity, voxel-based morphometry, magnetic resonance imaging

## Abstract

**Objective:**

White matter hyperintensities (WMHs) on magnetic resonance imaging (MRI) is frequently presumed to be secondary to cerebral small vessel disease (CSVD) and associated with cognitive decline. The cerebellum plays a key role in cognition and has dense connections with other brain regions. Thus, the aim of this study was to investigate if cerebellar abnormalities could occur in CSVD patients with WMHs and the possible association with cognitive performances.

**Methods:**

A total of 104 right-handed patients with WMHs were divided into the mild WMHs group (*n* = 39), moderate WMHs group (*n* = 37), and severe WMHs group (*n* = 28) according to the Fazekas scale, and 36 healthy controls were matched for sex ratio, age, education years, and acquired resting-state functional MRI. Analysis of voxel-based morphometry of gray matter volume (GMV) and seed-to-whole-brain functional connectivity (FC) was performed from the perspective of the cerebellum, and their correlations with neuropsychological variables were explored.

**Results:**

The analysis revealed a lower GMV in the bilateral cerebellum lobule VI and decreased FC between the left- and right-sided cerebellar lobule VI with the left anterior cingulate gyri in CSVD patients with WMHs. Both changes in structure and function were correlated with cognitive impairment in patients with WMHs.

**Conclusion:**

Our study revealed damaged GMV and FC in the cerebellum associated with cognitive impairment. This indicates that the cerebellum may play a key role in the modulation of cognitive function in CSVD patients with WMHs.

## Introduction

White matter hyperintensities (WMHs) is a state of chronic hypoperfusion in the white matter, which reflects the loss of axons and myelin, myelin pallor, and gliosis. It is described as hyperintense in the subcortical white matter displayed on T2-weighted MRI images and fluid-attenuated inversion recovery (FLAIR) images (Gebeily et al., 2014). Cerebral small vessel disease (CSVD), associated with the altered blood supply to the brain white matter, can lead to localized ischemic areas of necrosis and cavitation and cause WMHs (Petito et al., 1998). The prevalence of WMHs in the general population aged 60 to 90 years is nearly 70% in the Chinese population (Han et al., 2018). Moreover, there is growing evidence that the WMHs is imperative to cognitive dysfunction, particularly in executive function. Niels et al. reported that increasing severity of periventricular WMHs was specifically associated with impaired information processing speed and executive function (Prins et al., 2005; Smith et al., 2011; Yamanaka et al., 2019).

The cerebellum has long been regarded as critical for intact motor functioning (Koziol et al., 2014). Until recently, it has been accepted that it plays a significant role in cognitive processing supported by the posterior cerebellum, which is noteworthy (Smith et al., 2015a). Increasing numbers of studies have proven this. A meta-analysis revealed that patients with focal cerebellar lesions performed significantly worse on neuropsychological tests including phonological fluency, semantic fluency, Stroop test, block design test (WAIS-R), and visual memory (Ahmadian et al., 2019). Furthermore, lesions of the posterior lobe (lobules VI, VII, and possibly lobule IX) are reported to result in cerebellar cognitive affective syndrome characterized by impairments in executive function (Schmahmann, 2019).

Previous studies investigating the associations between WMHs on MRI and cognitive impairment have reported different results, with the most possible key role for prefrontal cortex connectivity with other (sub-)cortical regions (Chen et al., 2019). Moreover, structural and functional brain abnormalities in the cerebellum have also been recently identified in WMHs recently (Buckner, 2013). Studies have reported that both the number and volume of WMHs are correlated with changes in brain connectivity, especially in the cerebellum (Hoogendam et al., 2012; Smith et al., 2015b). Accumulating evidence has led to the growing recognition of the cerebellum’s role in cognitive function and its involvement in WMHs. However, in the vast majority of neuroimaging studies of WMHs, the cerebellum’s role in cognition has been dismissed or remains largely on the periphery in favor of cortico-centric models. Therefore, all these findings paved the way for our in-depth examination of the emerging role of the cerebellum in cognitive function in WMHs.

All these studies suggested that the cerebellum may play a vital role in cognition, and cerebellar involvement is described in several neurodegenerative diseases. For this reason, the aims of this study were to investigate if cerebellar abnormalities could occur in CSVD patients with WMHs and to investigate the possible association with cognitive performances. Therefore, we conducted voxel-based morphometry analysis (VBM) to explore cerebellar gray matter volume (GMV) and seed-to-whole-brain functional connectivity (FC) to investigate abnormalities of FC related to the cerebellum. Seed regions for resting-state analysis were selected based on GMV differences between the WMHs group and healthy controls (HC) group. Meanwhile, we also performed a correlation analysis between GMV as well as FC changes and cognitive performance in CSVD patients with WMHs.

## Materials and Methods

### Participants

This study included 104 patients with WMHs from the Department of Neurology of the First Affiliated Hospital of Anhui Medical University, Hefei, China. The inclusion criteria were as follows: (1) aged between 40 and 80 years and (2) visible WMHs on T2 fluid-attenuated inversion recovery (T2 FLAIR). The exclusion criteria were as follows: (1) intracranial and extracranial stenosis of >50%; (2) Trial of Org 10172 in Acute Stroke Treatment classification suggestive of cardiogenic stroke; (3) non-CSVD-related WMHs (e.g., multiple sclerosis); (4) mental disorders, alcohol addiction; (5) tumors; (6) intracranial hemorrhage; (7) significant hearing or visual impairment and physical movement disorders that prevented cooperation during cognitive testing; (8) language barrier; and (9) MRI contraindications or known claustrophobia. Periventricular hyperintensity (PVH) and deep white matter hyperintensity (DWMH) were scored separately using the four-point scale according to the Fazekas scale on FLAIR images (Fazekas et al., 1987). The PVH scores were categorized as follows: 0, absent; 1, caps or pencil-thin lining; 2, smooth “halo”; and 3, irregular PVH lesions extending into the deep white matter. DWMH scores were categorized as follows: 0, absence; 1, punctate foci; 2, beginning confluence of foci; and 3, large confluent areas. The Fazekas score is the sum of the PVH and DWMH scores. Patients with WMHs were divided into three groups based on their Fazekas scores. The mild WMHs group scored 1–2, the moderate WMHs group scored 3–4, and the severe WMHs group scored 5–6. Finally, we included 39 patients in the mild group, 37 patients in the moderate group, and 28 patients in the severe group.

Thirty-six controls (HC) were relatives of patients with WMHs and social recruits studied during the same period who matched the demographic data of the patients with WMHs, including age, gender, and years of education, and had no previous history of neurological diseases or mental illnesses. Imaging showed no white matter hyperintensities. The study was approved by the Ethics Committee of Anhui Medical University. All participants provided written informed consent before the study.

### Statistical Analyses

One-way analysis of variance was used to assess differences in age, education, and CSVD neuroimaging manifestations and differences between the neuropsychological test scores of the four groups (significant for *P* < 0.05), and the least significant difference was used for *post-hoc* analysis (significant for *P* < 0.05). The chi-squared test was used to determine gender differences in the four groups (*P* < 0.05).

### Magnetic Resonance Parameters

Structural and functional MRI were performed with a 3-T scanner (Discovery 750; GE Healthcare, Milwaukee, WI, United States) at the Information Science Center of the University of Science and Technology of China. During resting-state functional MRI (rs-fMRI) scanning, participants were instructed to keep their eyes closed, but not to fall asleep, and try to think of nothing in particular. A tight but comfortable foam padding was used to minimize head motion, and earplugs were used to reduce scanner noise. The parameters of the different sequences were set as follows: T2FLAIR: repetition time (TR) = 8,000 ms, echo time (TE) = 165 ms, TI = 2,000 ms, flip angle = 111°, matrix size = 512 × 512, field of view (FOV) = 256 mm × 256 mm, slice thickness = 5 mm, gap = 1 mm, and total slices = 20. Enhanced gradient echo T2 star-weighted angiography (ESWAN): TR = 52.189 ms, TE = 2.856 ms, flip angle = 12°, matrix size = 256 × 256, FOV = 220 mm × 220 mm, and slice thickness = 2 mm with no gap. Rs-fMRI: TR = 2,400 ms, TE = 30 ms, flip angle = 90°, matrix size = 64 × 64, FOV = 192 mm × 192 mm, slice thickness = 3 mm with no gap, and 46 continuous slices (one voxel = 3 mm × 3 mm × 3 mm).

Sagittal three-dimensional (3D) T1-weighted images were acquired using a brain volume (BRAVO) sequence with 188 slices (TR = 8.16 ms; TE = 3.18 ms; flip angle = 12°; FOV = 256 mm × 256 mm; slice thickness = 1 mm, no gap; voxel size = 1 mm × 1 mm × 1 mm).

### Neuropsychological Test

The neuropsychological scale of the Chinese Cerebral Small Vessel Disease Clinical Evaluation Study was used to evaluate the global cognitive function and individual cognitive functions of all participants (Cao et al., 2020). The Montreal Cognitive Assessment (MoCA) was used to measure global cognitive function (Nasreddine et al., 2005). Anxiety was assessed using the Generalized Anxiety Disorder-7 (GAD-7; Buckner, 2013). Depression symptoms were assessed using the Patient Health Questionnaire-9 (PHQ-9; Smarr and Keefer, 2011). The Chinese version of the Auditory Verbal Learning Test (AVLT) was used to evaluate memory function. The AVLT is composed of immediate memory, delay memory, and recognition memory function (Schoenberg et al., 2006). The Symbol Digit Modalities Test (SDMT) was used to evaluate information processing speed (Silva et al., 2018). The Digital Span test consists of two parts, forward and backward, and was used to evaluate the attention of the subjects (Yamamoto et al., 2011), and the Stroop Color-Word Test was used to evaluate executive function. The Trail Making Test (TMT) was also used to evaluate the executive and attention function (Selnes et al., 1991), and the Boston Naming Test was used to evaluate word-finding ability (Mack et al., 1992). The testers were all graduate or doctoral students in neurology who had passed the unified training.

### WMHs Volume

The WMHs volume was calculated using the UBO detector (Jiang et al., 2018)^1^, which is a cluster-based software. The calculation process was described in a previous study (Wen et al., 2009).

### Rs-fMRI Data Processing

All rs-fMRI data were preprocessed using the Data Processing Assistant for Resting-State Functional MR Imaging toolkit (Chao-Gan and Yu-Feng, 2010) (DPARSF; http://rfmri.org/content/dparsf) and statistical parametric mapping (SPM12; https://www.fil.ion.ucl.ac.uk/spm/software/spm12/). Preprocessing mainly includes the following. (1) The first five time points were removed to reduce the impact of the scanner during the initial scanning and facilitate the adaptation of the participants to the scanning environment. (2) The slice timing was used to correct the differences in acquisition time between layers of the volume. (3) Head correction was performed by removing participants with large head movements to reduce the effect on the result. (4) Space standardization was used to reduce the impact of human BRAVO and shape diversity. 3D T1 was used to register functional images for the spatial standardization of the participants in our study. A matrix was generated after registration and segmentation. The matrix data were applied to the functional images, which were used to register the participants from the functional to the standard space. (5) Smoothing was applied to reduce the incomplete effects of registration to ensure that the residuals were more consistent with the Gaussian distribution and improve the image signal-to-noise ratio. An 8-mm Gaussian kernel was chosen. (6) A detrending was applied at the end to reduce the effects of scanner heating. (7) Regression 24 Friston motion parameters, white matter high signal, and CSF were analyzed.

### VBM Analysis

Voxel-based morphometry analyses were performed to determine potential differences in the cerebellum between patients in the WMHs group and the HC group. T1-weighted anatomic images were preprocessed using the VBM8 toolbox in SPM8 (Statistical Parametric Mapping software). Each structural image was segmented into gray matter (GM), white matter, and cerebrospinal fluid using a fully automated algorithm within SPM8 and subsequently transformed into the Montreal Neurological Institute (MNI) space using diffeomorphic anatomical registration through exponentiated Lie algebra (DARTEL) normalization. Next, the normalized GM images were smoothed (FWHM = 8 mm) for statistical analyses. Finally, a one-way ANOVA was conducted with age, education, gender, and whole-brain GM volume as covariates on these normalized cerebellum images to determine structural differences. The Gaussian random field (GRF) correction (significant for voxel levels at *P* < 0.001 and cluster at *P* < 0.05) was used for correction. Statistical analysis for saving masks was performed using the Data Processing and Analysis of Brain Imaging toolbox (Wu et al., 2020).

### Rs-FC Calculation

We extracted the right-sided cerebellum lobule VI and the left-sided cerebellum lobule VI as the seed areas for resting-state FC analyses. The rs-FC between the mean time series of the seed and the time series of each voxel in the remainder of the GM was calculated using Pearson’s correlation coefficient of the time course of each seed. To facilitate the normality of the data distribution, the correlation coefficients were converted to *z*-values using Fisher’s *r*-to-*z* transformation.

Based on a GM template, a one-way analysis of covariance was used to assess the differences between the functional connectivities of the four groups. Age, education, GM volume, and gender were considered as covariates. Statistical analysis was performed voxel-wise using DPARSF and SPM software. Multiple comparisons during the analyses were corrected using the GRF (significant for voxel levels at *P* < 0.001 and cluster at *P* < 0.05).

### Correlation Analysis

We performed a Pearson correlation analysis between each participant’s volume of the cerebellum and neuropsychological tests and between each participant’s FC and neuropsychological tests in significant regions to further explore whether neuroimaging indices were related to cognitive functions (significant for *P* < 0.05).

## Results

### Demographic and Clinical Characteristics

There were no significant differences between the ages, years of education, and gender distributions of the HC and WMHs groups. The proportion of patients with hypertension was higher than that of HC, but there were no significant differences in the four groups in terms of diabetes, hyperlipidemia, smoking history, and drinking history. Demographic information and CSVD marker information are displayed in Table 1. Regarding the severity of WMHs, the quantitative assessment method we used was in good agreement with the Fazekas score (*P* < 0.001, *r* = 0.499). The distribution of lacunes and microbleed lesions is shown in Table 2. However, several participants did not complete the ESWAN sequence scan for specific reasons (HC group: 5 participants; mild WMHs group: 13 patients; moderate WMHs group: 7 patients; and severe WMHs group: 7 patients). Besides, neuropsychological tests of the participants in the four groups are shown in Table 3.

**TABLE 1:** Demographic and cerebral small vessel disease neuroimaging manifestations of participants in the four groups [mean (SD)].

	**HC (*n* = 36)**	**Mild WMHs (*n* = 39)**	**Moderate WMHs (*n* = 37)**	**Severe WMHs (*n* = 28)**	***F*/*χ*^2^**	***P***
Age (years)	60.58 (5.98)	63.77 (8.23)	65.08 (10.03)	65.04 (6.90)	2.424	0.068
Female	18	16	14	16	3.011	0.390
Education (years)	9.31 (3.32)	8.41 (4.16)	8.00 (3.84)	7.75 (3.88)	1.083	0.359
Hypertension	8	21	22	21	19.568	<0.001***
Diabetes	2	8	7	4	3.905	0.272
Hyperlipidemia	8	10	9	8	0.356	0.949
Smoking history	9	18	15	11	3.808	0.283
Drinking history	12	9	16	12	4.346	0.226
Fazekas	0.00 (0.00)^*a,b,c*^	1.64 (0.49)^*a,d,e*^	3.59 (0.50)^*b,d,f*^	5.43 (0.50)^*c,e,f*^	958.855	<0.001***
WMHs volume	/	11,457.35 (12,493.05)^*d,e*^	18,067.56 (10,873.84)^*d,f*^	30,658.38 (12,033.91)^*e,f*^	21.696	<0.001***
Lacunes	0.00 (0.00)^*a,b,c*^	0.51 (0.91)^*a,d*^	0.89 (1.29)^*b,d*^	0.79 (1.23)^*c*^	5.626	0.001**
Microbleeds	0.31 (0.59)^*c*^	2.26 (3.61) ^*e*^	1.50 (2.35)^*f*^	6.29 (14.96)^*c,e,f*^	3.389	0.021**

Volume is in cubic millimeters. HC, healthy controls; WMHs, white matter hyperintensities; and SD, standard deviation. ^*a*^Healthy control group vs. mild WMHs group significantly different (*P* < 0.05). ^*b*^Healthy control group vs. moderate WMHs group significantly different (*P* < 0.05). ^*c*^Healthy control group vs. severe WMHs group significantly different (*P* < 0.05). ^*d*^Mild WMHs group vs. moderate WMHs group significantly different (*P* < 0.05). ^*e*^Mild WMHs group vs. severe WMHs group significantly different (*P* < 0.05). ^*f*^Moderate WMHs group vs. severe WMHs group significantly different (*P* < 0.05). ***Significant at 0.001 level, **significant at 0.01 level.

**TABLE 2:** Distribution of neuroimaging manifestations in HC and patients with WMHs [mean (SD)].

	**Lacunes**				**CMBs**			
	**HC (*n* = 36)**	**Mild WMHs (*n* = 39)**	**Moderate WMHs (*n* = 37)**	**Severe WMHs (*n* = 28)**	**HC (*n* = 32)**	**Mild WMHs (*n* = 27)**	**Moderate WMHs (*n* = 30)**	**Severe WMHs (*n* = 21)**
**Subcortical**								
Frontal	0.00 (0.00)	0.18 (0.51)	0.11 (0.39)	0.14 (0.45)	0.03 (0.18)	0.11 (0.32)	0.13 (0.57)	0.33 (0.80)
Parietal	0.00 (0.00)	0.03 (0.16)	0.11 (0.39)	0.00 (0.00)	0.09 (0.30)	0.19 (0.68)	0.13 (0.35)	0.33 (0.91)
Occipital	0.00 (0.00)	0.00 (0.00)	0.05 (0.23)	0.04 (0.19)	0.03 (0.18)	0.07 (0.27)	0.07 (0.25)	0.57 (1.36)
Temporal	0.00 (0.00)	0.08 (0.27)	0.05 (0.33)	0.21 (0.50)	0.03 (0.18)	0.41 (1.15)	0.23 (0.63)	1.48 (3.12)
Any subcortical	0.00 (0.00)	0.03 (0.16)	0.08 (0.28)	0.11 (0.31)	0.00 (0.00)	0.04 (0.19)	0.00 (0.00)	0.19 (0.87)
**Deep**								
Basal ganglia	0.00 (0.00)	0.18 (0.51)	0.38 (0.79)	0.18 (0.61)	0.03 (0.18)	0.22 (0.51)	0.17 (0.38)	0.71 (1.23)
Thalamus	0.00 (0.00)	0.00 (0.00)	0.03 (0.16)	0.00 (0.00)	0.00 (0.00)	0.07 (0.27)	0.17 (0.46)	0.43 (1.33)
Internal capsule	0.00 (0.00)	0.00 (0.00)	0.03 (0.16)	0.00 (0.00)	0.03 (0.18)	0.44 (0.84)	0.23 (0.77)	0.62 (1.72)
Any deep	0.00 (0.00)	0.00 (0.00)	0.03 (0.16)	0.11 (0.31)	0.03 (0.18)	0.11 (0.32)	0.20 (0.66)	0.24 (0.89)
**Infratentorial**								
Cerebellum	0.00 (0.00)	0.00 (0.00)	0.03 (0.16)	0.00 (0.00)	0.03 (0.18)	0.15 (0.36)	0.13 (0.35)	0.86 (3.50)
Pons	0.00 (0.00)	0.03 (0.16)	0.00 (0.00)	0.00 (0.00)	0.00 (0.00)	0.11 (0.42)	0.03 (0.18)	0.14 (0.48)
Mesencephalon	0.00 (0.00)	0.00 (0.00)	0.00 (0.00)	0.00 (0.00)	0.00 (0.00)	0.15 (0.46)	0.03 (0.18)	0.14 (0.65)
Any	0.00 (0.00)	0.00 (0.00)	0.00 (0.00)	0.00 (0.00)	0.00 (0.00)	0.04 (0.19)	0.00 (0.00)	0.29 (1.10)
Any	0.00 (0.00)	0.51 (0.91)	0.89 (1.29)	0.79 (1.23)	0.31 (0.59)	2.11 (3.53)	1.53 (2.36)	6.33 (14.96)

*SD, standard deviation; CMBs, cerebral microbleeds.*

**TABLE 3:** Neuropsychological tests of participants in the four groups [mean (SD)].

	**HC (*n* = 36)**	**Mild WMHs (*n* = 39)**	**Moderate WMHs (*n* = 37)**	**Severe WMHs (*n* = 28)**	***F*/*χ*^2^**	***P***
MoCA	22.33 (3.09)^*c*^	21.16 (4.32)^*d*^	21.06 (3.79)^*e*^	17.81 (4.91)^*c, d, e*^	6.850	<0.001***
AVLT-study	8.15 (1.59)^*c*^	7.63 (2.24)^*d*^	7.31 (1.52)	6.55 (2.00)^*c, d*^	3.776	0.012*
AVLT-immediate	9.40 (2.30)^*c*^	8.17 (3.84)^*d*^	7.88 (2.65)^*e*^	6.17 (3.27)^*c, d, e*^	5.320	0.002**
AVLT-delay	9.06 (2.41)^b, c^	7.95 (3.76)	7.31 (2.67)^*b*^	6.50 (3.13)^*c*^	3.731	0.013*
AVLT-recognition	13.63 (1.33)^*c*^	13.53 (3.68)^*d*^	13.16 (1.87)^*e*^	11.13 (3.62)^*c, d, e*^	4.734	0.004**
TMT-A	63.34 (24.76)^*a, b, c*^	80.72 (33.12)^*a, d*^	79.12 (33.73)^*b, e*^	105.82 (37.72)^*c, d, e*^	8.233	<0.001***
TMT-B	129.65 (44.44)^*c*^	155.35 (70.69)^*d*^	158.16 (73.16)^*e*^	207.55 (81.24)^*c, d, e*^	6.301	0.001**
BNT	13.94 (0.98)^*a, b, c*^	12.89 (1.54)^*a*^	13.25 (1.68)^*b*^	12.74 (1.40)^*c*^	4.797	0.003**
PHQ-9	3.31 (4.78)^*c, d*^	3.74 (4.56)^*d*^	5.00 (4.62)	7.39 (5.81)^*c, d*^	4.272	0.006**
GAD-7	2.40 (3.24)^*c, d*^	2.61 (4.10)^*d*^	2.81 (3.43)^*e*^	5.29 (5.48)^*c, d, e*^	3.282	0.023*

HC, healthy controls; WMHs, white matter hyperintensities; SD, standard deviation; MoCA, Montreal Cognitive Assessment; PHQ, Patient Health Questionnaire; GAD, generalized anxiety disorder; AVLT, Chinese Auditory Learning Test; TMT, Trial Making Test; and BNT, Boston Naming Test. ^*a*^Healthy control group vs. mild WMHs group significantly different (*P* < 0.05). ^*b*^Healthy control group vs. moderate WMHs group significantly different (*P* < 0.05). ^*c*^Healthy control group vs. severe WMHs group significantly different (*P* < 0.05). ^*d*^Mild WMHs group vs. severe WMHs group significantly different (*P* < 0.05). ^*e*^Moderate WMHs group vs. severe WMHs group significantly different (*P* < 0.05). ***Significant at 0.001 level, **significant at 0.01 level, and *significant at 0.05 level (two tailed).

### Group Comparison of Cerebellar Atrophy

**FIGURE 1:**
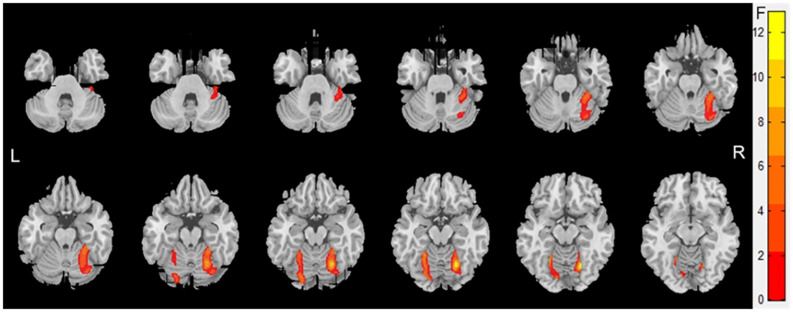
Voxel-based morphometry showing gray matter loss in the cerebellar subregions for patients with White matter hyperintensities (WMHs) in comparison with controls. Areas of significant gray matter loss (red) included the cerebellar subregions right-sided lobule VI [voxel size: 2,106; peak MNI coordinates: *x* = 22.5, *y* = –63, and *z* = –15; Gaussian random field (GRF) for voxel levels at *P* < 0.001 and cluster at *P* < 0.05] and left-sided lobule VI (voxel size: 703; peak MNI coordinates: *x* = –22.5, *y* = –54, and *z* = –15; GRF for voxel levels at *P* < 0.001 and cluster at *P* < 0.05), for patients with WMHs vs. control subjects.

**FIGURE 2:**
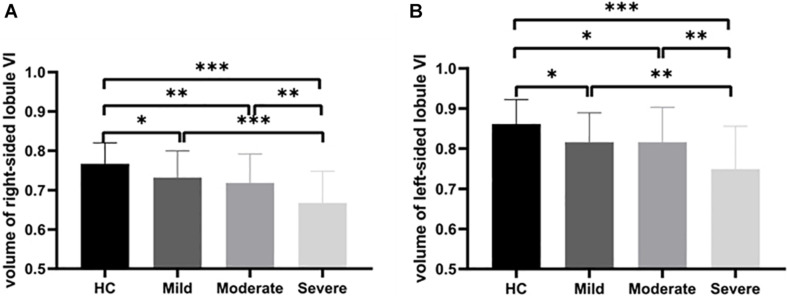
Group comparison of cerebellar atrophy. Volume contrasts within two subregions of the cerebellum across four groups. *Post-hoc* analysis of the brain regions with significant differences among the four groups. **(A)** The gray matter volume (GMV) of the right-sided cerebellum lobule VI showing atrophy in WMHs groups. **(B)** The GMV of the left-sided cerebellum lobule VI showing atrophy in WMHs groups. ^∗∗∗^*P* < 0.001, ^∗∗^*P* < 0.01, and ^∗^*P* < 0.05.

Atrophy in the cerebellar subregions was apparent in patients with WMHs relative to HCs. Specifically, two large contiguous clusters were identified shown in Figure 1. One cluster included cerebellar subregions right-sided lobule VI (voxel size: 1961; peak MNI coordinates: *x* = 22.5, *y* = −63, *z* = −15; GRF for voxel levels at *P* < 0.001 and cluster at *P* < 0.05) and another included left-sided lobule VI (voxel size: 622; peak MNI coordinates: *x* = −12, *y* = −82.5, *z* = −13.5; GRF for voxel levels at *P* < 0.001 and cluster at *P* < 0.05). The volumes of the two ROIs were significantly higher in the HC group than in the patients with WMHs and gradually decreased in patients with WMHs from the mild to severe group (shown in Figure 2). The severe group also showed reduced volume both in right-sided lobule VI and left-sided lobule VI compared to the mild and moderate groups.

**FIGURE 3:**
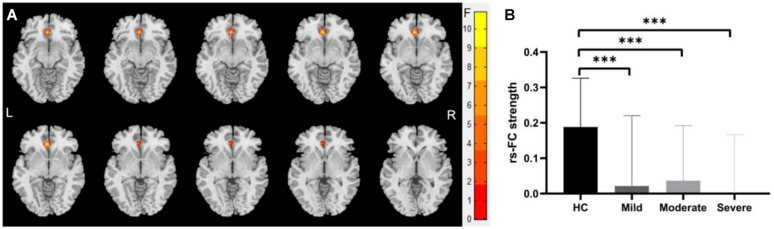
Differences of functional connectivity (FC) between groups at the right-sided cerebellum lobule VI—whole brain level. **(A)** Significant differences were observed between patients with WMHs and healthy controls (HC) in the FC between the right-sided cerebellum lobule VI with the left anterior cingulate gyri (voxel size: 39; peak MNI coordinates: *x* = –3, *y* = 33, and *z* = –6; GRF for voxel levels at *P* < 0.001 and cluster at *P* < 0.05). **(B)** The functional connectivity of the right-sided cerebellum lobule VI with the left anterior cingulate gyri was significantly decreased in patients with WMHs. ^∗∗∗^*P* < 0.001.

We identified significantly decreased functional connection between the left-sided cerebellum lobule VI with the left anterior cingulate gyrus (voxel size: 39; peak MNI coordinates: *x* = −3, *y* = 33, *z* = −6; cluster-level GRF for voxel levels at *P* < 0.001 and cluster at *P* < 0.05) in mild, moderate, and severe WMHs groups compared with HCs (shown in Figure 3).

Correlation analyses revealed that the volume of the left-sided cerebellum lobule VI was negatively correlated with TMT-A test (*P* = 0.026, *r* = −0.361) and TMT-B test (*P* = 0.027, *r* = −0.363) in the mild group, and positively correlated with AVLT-study test (*P* < 0.001, *r* = 0.670), AVLT-immediate test (*P* = 0.002, *r* = 0.601), AVLT-delay test (*P* = 0.001, *r* = 0.644), AVLT-recognition test (*P* = 0.033, *r* = 0.437) results in the severe group. The volume of the right-sided cerebellum lobule VI was positively correlated with the AVLT-immediate test (*P* = 0.022, *r* = 0.398), AVLT-delay test (*P* = 0.015, *r* = 0.428) results in the moderate group, and positively correlated with the AVLT-study test (*P* = 0.001, *r* = 0.607), AVLT-immediate test (*P* < 0.001, *r* = 0.757), AVLT-delay test (*P* < 0.001, *r* = 0.800), AVLT-recognition test (*P* < 0.001, *r* = 0.628) in the severe group.

## Discussion

In this study, we investigated the alterations in the volume and FC in the cerebellum due to WMHs and evaluated their relevance to cognitive function. Regarding imaging data, for the group difference, we have shown a lower volume in cerebellum lobule VI and lower FC between the left and right cerebellar lobule VI with the left anterior cingulate gyri in patients with WMHs. Moreover, both were closely related to WMHs scores and cognitive functions.

Recently, studies have demonstrated an increasing interest in elucidating the pattern of cerebellar atrophy across diseases and revealed its critical role in many neurodegenerative diseases (Guo et al., 2016). Although WMHs is commonly observed in cerebral white matter, CSVD-associated lesions can also occur in GM. We found cerebellar atrophy in lobule VI in WMHs patients, which is in line with numerous studies of neurodegenerative diseases (Tan et al., 2014). As for Alzheimer’s disease (AD), atrophy of the posterior cerebellar regions was identified (Guo et al., 2016), and in Parkinson’s disease (O’Callaghan et al., 2016), direct pathological change in the cerebellum, including atrophy, has also been confirmed (Borghammer et al., 2010). However, the functional consequences of GM loss in the cerebellum are not uniform across these studies. Cerebellar lobule VI has been implemented in executive functions that dovetail with the predominant cognitive impairment characteristic of WMHs (Habas et al., 2009). This is supported by our study that found a correlation between cerebellar lobule VI atrophy and cognitive decline in WMHs.

Reduced connectivity of the cerebellum lobule VI to the left anterior cingulate gyri owing to white matter hyperintensities was also found in this study. Observed gray matter loss in cerebellum lobule VI may drive this change in FC, and the WMHs can also disrupt white matter tracts or U-fibers that mediate cortical–cortical or cortical–subcortical connections (Ward et al., 2015). Furthermore, connectivity studies have confirmed that the posterior cerebellum has strong connections with the prelimbic, orbitofrontal, and anterior cingulate cortex, which provide anatomical substrates for our results (Badura et al., 2018). Aligning with our results, Schaefer et al. reported connectivity changes in cerebellar regions that are connected to frontoparietal cognitive networks (lobules crus II and VIIb) in patients with CSVD (Schaefer et al., 2014). Another study revealed that both the number and volume of WMHs were correlated with decreased FC of the cerebellum (Kerkovsky et al., 2019). Our findings provide further support for cerebellar involvement in WMHs. Despite the difficulties in interpreting WMHs pathology, the fact that atrophy and impaired FC correlated with WMHs severity observed in our study suggested that cerebellar changes should be at least partly related to the basic pathological process of WMHs.

The existing studies have shown that in patients with WMHs, memory, processing speed, and executive function impairments exist compared with HCs, and structural and functional brain abnormalities in the cerebellum have also been reported (Buckner, 2013; Kaskikallio et al., 2019). However, the association between cognitive decline and cerebellum in patients with WMHs is rarely discussed. The cerebellum has traditionally been thought to contribute mainly to motor coordination (Ito, 2000; Gao et al., 2012). However, increasing evidence has demonstrated that the cerebellum also plays an important role in cognitive processing, and memory and executive functions are mostly localized in the posterior cerebellum (E et al., 2014), which is supported by many researchers. Functional MRI evidence found cerebellar activation is a consistent finding with memory tasks predominantly in the posterior lobe of the cerebellum (Zacharia and Eslinger, 2019). Schmahmann found that lesions of the posterior lobe of the cerebellum were particularly important in impairments in executive function (Zhuang et al., 2017; Schmahmann, 2019). Researchers suspect that the most likely route for cerebellar contribution to cognition is *via* interactions with the neocortex (Wagner and Luo, 2020). This means that damage to the posterior lobe of the cerebellum and its connections may potentially degrade and disconnect cognition subserved by it. In line with previous studies, both atrophy and hypoconnectivity of cerebellum lobule VI were found to be related to cognitive decline in WMHs in our study, suggesting that the cerebellum with its influence on the cingulate cortex is responsible for cognitive decline in patients with WMHs. This is particularly relevant because the cerebellum’s ability to work full time likely depends on its internal integrity and the integrity of its cortical connections, and local gray matter change in cerebellum lobule VI, or its functional connections (the cingulate), might influence cerebellar activity.

Several limitations of this study should be addressed. First, the sample size was relatively small, and therefore, our findings require validation in a larger cohort. Second, this cross-sectional study only provides correlational but no causal associations that need to be approached by longitudinal study designs. Third, it is impossible to eliminate the concomitant structural changes of WMHs, such as lacunes, although the lacunes were much less severe than WMHs in the current study. Further studies using a prospective design are needed to address these issues.

In conclusion, this study demonstrated that CSVD patients with WMHs display a lower bilateral GMV and a decreased FC in specific subregions of the cerebellum related to cognitive functions (VI). Moreover, correlations exist between these brain alterations and specific neurocognitive functions including memory and executive function. Together, it is quite a novelty to demonstrate that cerebellar abnormalities could occur in CSVD patients with WMHs and to introduce its unique contribution to cognitive functions, which led to the growing recognition of the cerebellum’s role in CSVD patients with WMHs.

## Data Availability Statement

The raw data supporting the conclusions of this article will be made available by the authors, without undue reservation.

## Ethics Statement

The studies involving human participants were reviewed and approved by Ethics Committee of the First Affiliated Hospital of Anhui Medical University. The patients/participants provided their written informed consent to participate in this study. Written informed consent was obtained from the individual(s) for the publication of any potentially identifiable images or data included in this article.

## Author Contributions

SC and JN: performed the analysis and wrote the manuscript. KW: made substantial contribution to the conception of the work. KW, YT, and QW: designed the work. CC, XW, and YL: conducted acquisition and analysis. YM and BD: interpreted the data for the work. KW, YT, and QW: gave final approval of the version to be published and agreed to be accountable for all aspects of the work. All authors contributed to the article and approved the submitted version.

## Conflict of Interest

The authors declare that the research was conducted in the absence of any commercial or financial relationships that could be construed as a potential conflict of interest.

## Footnotes

1 https://cheba.unsw.edu.au/group/neuroimagingpipeline
